# Outcomes of Neonatal Candidiasis: The Impact of Delayed Initiation of Antifungal Therapy

**DOI:** 10.1155/2011/813871

**Published:** 2011-11-03

**Authors:** Heather Cahan, Jaime G. Deville

**Affiliations:** ^1^Department of Pediatrics, Cedars-Sinai Medical Center, 8700 Beverly Boulevard, Los Angeles, CA 90048, USA; ^2^Division of Pediatric Infectious Diseases, Department of Pediatrics, UCLA School of Medicine, University of California, 10833 Le Conte Avenue, MDCC 22-442, Los Angeles, CA 90095-1752, USA

## Abstract

*Objective/Methods*. To determine the outcomes of invasive neonatal candidiasis before institution of routine antifungal prophylaxis, we conducted a retrospective review of cases of invasive candidiasis in newborns in a referral-based neonatal intensive care unit located in a single tertiary academic center between January 1998 and December 2002. *Results*. Sixty-three newborns with invasive neonatal candidiasis were identified. Overall mortality rate was 35%. Virtually every infant had a central venous catheter (CVC), required mechanical ventilation and previous administration of antibacterial agents. Delayed institution of antifungal therapy was associated with increased mortality. In addition, length of hospitalization, duration of prior antibacterial therapy, mechanical ventilation, and CVC use, as well as evidence of end-organ disease, were associated with an adverse outcome. *Conclusions*. Reliance on available laboratory tools in cases of invasive neonatal candidiasis can result in delayed diagnosis and increased mortality. A risk-factor-based approach to empirical treatment could be justified in this setting.

## 1. Introduction

Invasive fungal infections are a major problem in the neonatal intensive care unit (NICU). *Candida *species are as the fourth most common cause of late-onset infections in the neonatal intensive care unit (NICU) and are responsible for considerable morbidity and mortality [1]. About 12% of all neonatal infections are due to *Candida* species [2–4]; cases increased 10-fold between 1981 and 1995 [5, 6]. Reported mortality rates for infants with invasive neonatal candidiasis are between 25 and 50%, compared to an overall mortality of 4.7% for all NICU admissions [5].

Major risk factors associated with invasive neonatal candidiasis include extreme prematurity, prolonged central venous catheterization, and prolonged use of broad-spectrum antibacterial agents [7–12]. 

Blood cultures are still considered as the gold standard for the diagnosis of invasive neonatal candidiasis. However, when compared with bacterial pathogens, their sensitivity is less than ideal. In a large report of autopsy-proven invasive candidiasis [13], the majority of cases lacked a positive blood culture. In a cohort of 41 patients with hematologic malignancies and autopsy-proven candidiasis, 28% of those who had single-organ involvement had a previous positive blood culture. When at least 3 organs were found to be infected in postmortem examination, sensitivity of blood cultures increased to 78% [14]. The blood volumes necessary to optimize organism recovery are not easily attainable in neonates [15, 16]. Therefore, relying solely in blood cultures could underestimate the true incidence of invasive neonatal candidiasis. The absence of a fungal pathogen isolated in a blood culture may lead to underdiagnosis of invasive neonatal candidiasis, and in turn, to a delay in treatment institution, which could contribute to the high mortality observed in previous studies [1, 2, 9]. 

We assessed for the effect of delayed recognition and treatment of invasive neonatal candidiasis and for risk factors associated with mortality in a tertiary care NICU over the five-year period before the institution of routine fluconazole prophylaxis in our NICU.

## 2. Materials and Methods

### 2.1. Study Design

We conducted a retrospective cohort study of all cases of culture-proven invasive neonatal candidiasis admitted to the NICU at the UCLA Medical Center between January 1, 1998 and December 31, 2002.

### 2.2. Population

Study participants were identified from microbiology records of all infants admitted to the NICU with a positive fungal culture. Cases of invasive neonatal candidiasis were defined as those that fulfilled the following criteria (1) + (2a) or (1) + (2b):

1. a clinical picture compatible with fungal sepsis, defined by patients meeting at least 5 criteria [17]: admission to an NICU with high rate of candidemia, history of broad-spectrum antibiotic coverage, use of a third-generation cephalosporin negative bacterial blood culture results, a falling platelet count, use of systemic steroids, NPO, need for mechanical ventilation, and cardiovascular instability requiring pressor support;
2. a fungal pathogen isolated from blood culture either from a line or peripheral site, urine by bladder catheterization, peritoneal fluid by paracentesis, pleural fluid by pleurocentesis;
3. when the only fungal isolate came from a deep aspirate from an endotracheal tube, patients had to fulfill clinical criteria for sepsis *and* all 4 of the following clinical criteria for pneumonia (radiographic changes, need for increase in mechanical ventilation, increase in tracheal secretions, abnormal peripheral white blood cell count and/or bandemia).

The analysis was conducted comparing survivors with nonsurvivors. In cases of multiple fungal infections in a single patient, data was analyzed from the final episode in order to evaluate the primary outcome. Survival was defined as either discharge to home or transfer to a lower level of care.

### 2.3. Data

Medical records were reviewed, and information was obtained on gender, birth weight, gestational age, underlying illness, hospital course, invasive procedures, outcome, use of anti-infective agents, and bacterial infections.

### 2.4. Statistics

Data are presented descriptively for demographics, culture location and species of fungus responsible for infection. Student's *t*-test was used to compare single variable differences between mortality and survival to discharge groups. Multivariate analysis was used to identify significant associations of risk factors. Discrete data was compared by Chi-square or Fisher exact test.

## 3. Results

Between January 1, 1998 and December 31, 2002, there were 1686 admissions to the UCLA NICU. There were 157 deaths, for an overall mortality rate of 9.3%. We identified a total of 253 patients with a positive fungal culture. From these, 63 patients met our definition for invasive neonatal candidiasis, for a prevalence of 37 cases/1000 admissions. 22 of the 63 infants expired, for an overall mortality of 35%.

The mean gestational age (GA) of both the infants who expired and survived invasive neonatal candidiasis was 30 weeks (range: 23–40 weeks). Mean birth weight (BW) was 1628 grams (range: 409–4470) in patients who expired and 1741 grams (range 486–3800) in survivors. In the survivor group, 10 patients (24%) had GA between 23 and 25 weeks and 13 patients (32%) had GA of 26–34 weeks, compared with 7 (31%) and 4 (18%), respectively, in the mortality group.

10 survivors (24%) had congenital heart disease, compared with 5 (23%) infants in the mortality group. 3 survivors (7%) had other major congenital anomalies, compared with 3 nonsurvivors (14%). 5 survivors (12%) and 3 nonsurvivors (14%) were diagnosed with chromosomal anomalies. There were no significant differences in primary diagnoses between survivor and mortality groups. All but one of the 63 patients received mechanical ventilation and antibacterial agents. All patients had central venous catheters placed. A previous, culture-proven bacterial infection was identified in 27 survivors (66%) and 18 nonsurvivors (82%) (*P* = 0.25).

Demographics and antibacterial use are shown in Tables 1 and 2. Both groups differed significantly in mean number of positive fungal cultures, days with central venous catheters, mean platelet count nadir, days in NICU before invasive fungal infection, days on ventilator (both total and prior to fungal infection), previous use of a third-generation cephalosporin, and days of treatment with intravenous antibacterial agents prior to fungal infections. Mean duration of positive cultures, GA, birth weight, total ICU days, and days of antifungal therapy were comparable between those infants who expired and those who survived until discharge. 

The most common isolates identified were *Candida albicans* (57%) and *Candida parapsilosis* (27%), followed by *Candida tropicalis* (5%),* Candida lusitaniae *(3%),* Candida glabrata *(2%), and* Malassezia furfur *(2%). An unidentified yeast was isolated in 2 cases (3%). 

We did not find an association between fungal species and outcome of invasive fungal infections. We did not observe a significant difference in culture sites between the survivor and mortality groups (Table 3). Multivariate analysis demonstrated a higher risk of mortality with increasing number of positive fungal cultures as well as the number of days of antibiotic use prior to the fungal infection (*P* = 0.005 and 0.009, resp.).

Our population included 23 patient with blood isolates; of these, 15 had the CVC removed after the positive blood culture, 11/15 (73%) survived. 3 of 8 (38%) patients in which the CVC was not removed survived (*P* = 0.09).

Thirty-three patients had further diagnostic studies performed (head/cardiac/renal/abdominal ultrasound or ophthalmologic exam). Of these, 8 (24%) had evidence of invasive fungal disease. Of the 8 infants with end-organ evidence of fungal disease, 3 had only blood cultures positive and 5 had positive fungal cultures at multiple sites. 6 of these 8 infants expired (75%). In contrast, 6/25 (24%) patients who had no end-organ evidence of fungal disease expired (*P* < 0.01). Of these, 9 patients had multiple positive sites; in 7 the only source was urine, in 4 peritoneal fluid, 3 had only positive blood cultures, and 2 only tracheal cultures.

Seven of the 25 patients who expired (28%) underwent postmortem examination. 5 of these 7 had autopsy evidence of invasive fungal disease and a positive postmortem culture. Two patients had ischemic colitis and *Candida* peritonitis. They were 25- and 26-week premature infants, respectively, and each had treatment started 4 days after the first positive culture. One patient had *Candida albicans* isolated from blood, urine, and peritoneal cultures. No radiographic evaluation was done. The second patient had negative renal and abdominal ultrasounds obtained after peritoneal fluid yielded *Candida parapsilosis*. All other sites were negative in this infant. The third patient was a full-term infant with congenital heart disease who had autopsy evidence of *Candida* sepsis, with involvement of the heart, liver, and kidneys. Treatment was begun 3 days after blood cultures were positive for *Candida albicans*. No other sites were positive, and no radiologic evaluation was undertaken. The fourth patient was a 24-week premature infant who had renal mycetomas on autopsy. This patient had many negative blood and urine fungal cultures; the only positive culture isolated came from a tracheal aspirate which was positive for *Candida lusitaniae*. Treatment was begun 4 days after the first isolate was identified. One baby had gastroschisis and a peritoneal culture with *Candida albicans*. Microabscesses were identified in postmortem examination of the peritoneal cavity; however, postmortem cultures were negative. Treatment was initiated 4 days after isolation of the organism. No end-organ evaluation was done. 2 patients had negative autopsy culture findings; both were 24-week premature infants. One had *Candida parapsilosis* in tracheal aspirates, and the other had had *Candida parapsilosis* in tracheal aspirates and urine. 

We found a significant association between delay of antifungal administration and mortality. Median time to initiation of treatment after the first positive fungal culture was obtained was 4 days in the mortality group and 2 days in the survivor group (*P* = 0.004). When we analyzed the relationship between mortality and the interval from the day the first positive culture was drawn and the day that antifungal therapy was started, we found mortality rates of 22.5% (7/30), 40% (6/15), and 54% (7/13) when those intervals were 0–2 days, 2–7 days, and >7 days, respectively. Each 24-hour delay in the initiation of antifungal therapy was associated with a 10.9% increase in odds of mortality (OR 1.109, 95% CI: 1.003–1.226). When we exclude patients in which the only positive culture was obtained by tracheal aspirate, median times to treatment initiation are 4 days for the mortality group and 1 day for survivors (*P* = 0.02). Time from culture to treatment was similar in patients without a blood fungal isolate and fungemic patients. Similarly, we did not observe a significant difference in the distribution of culture sites in those patients in which therapy was delayed over 48 hours when compared with those infants who received antifungals earlier.

Fifty-eight of 63 patients (92%) received systemic antifungal therapy with either amphotericin B deoxycholate or amphotericin B lipid complex. Of the 5 patients who did not receive therapy, 3 survived, and two expired. Three had *Candida albicans*, and 2 had *Candida parapsilosis*. Their GA ranged from 25 to 40 weeks, and the diagnoses were as follows: chromosomal anomalies in one baby, prematurity in two infants, congenital heart disease in one patient, and noncardiac congenital anomalies in one subject. The source of isolates in the patients who expired was tracheal only in one patient and urine plus tracheal aspirate in one infant. In survivors, isolates came from peritoneal fluid in two infants and urine in one patient.

## 4. Discussion

We believe that the observation of increased mortality in patients where a longer interval ensued between obtaining cultures and stating antifungal therapy is important. Although certainly not surprising, an association between delay in initiation of antifungal therapy and mortality has not been documented previously in the literature. In severe infections, one can expect a more favorable outcome when therapy is promptly instituted. However, there is an inherent delay between the clinical presentation of sepsis, when cultures are presumably obtained, and the availability of results. A study by Schelonka and Moser [18] suggested that candidemia can be detected within 72 hours. A septic-appearing newborn routinely receives antibacterial agents at the time of clinical presentation. In contrast, many clinicians will only start antifungal therapy when a positive culture is reported or when the patient fails to improve after a period of antibacterial agents. In a retrospective study, Makhoul et al. [6] reported 49 cases of neonatal candidemia over a 10-year period, with no deaths. This was attributed to a policy of starting empiric antifungal therapy immediately after obtaining cultures in neonates with risk factors for fungal sepsis, instead of waiting for culture results. Benjamin Jr. et al. [19] evaluated data from a multicenter cohort to develop a model to provide guidance to neonatologists as to when to consider empirical antifungal therapy. 

We did not identify an association between culture site and therapy delay, which could potentially result in a clinician bias against nonblood isolates. Moreover, we did not observe a difference in mortality rates by culture sites. Patients in whom the only source for the fungal pathogen was a tracheal culture had a similar outcome to the remainder of the study population. Tracheal cultures are interpreted by many clinicians with skepticism, due to a concern about a perceived poor correlation between tracheal isolate and lower respiratory tract pathogens. This in turn could lead to a delay in initiating antifungal therapy. Several studies have found correlation between isolation of *Candida* in tracheal cultures and its presence in lung parenchyma or the subsequent risk of candidemia in neonates [20–22]. In a septic-appearing newborn with findings of clinical pneumonia, when a bacterial pathogen is not found, or when empiric antibacterial therapy fails to create a clinical response, one may consider that the presence of a fungal isolate in a tracheal aspirate should be considered a true pathogen, and specific therapy should be instituted. 

The patients who died had onset of infection at a mean of 69 days versus 33 days in the survivors. This conflicts with data presented previously from a review of a private practice database [23], which reported that a younger age at the time of infection is associated with mortality. This could be due to the inherent selection bias in patient complexity observed in large, tertiary referral institutions, when compared with community-based practices. 

Although *Candida albicans* was the most common isolate recovered, our data confirm the emergence of non-*albicans* species as major causes of invasive neonatal candidiasis [1, 2]. We did not find a correlation between fungal species and mortality. In a study of 45 cases of neonatal candidiasis over a 10-year period, Faix [24] reported that 7 of 29 infants with *Candida albicans *died, compared to no deaths in 16 infants with* Candida parapsilosis.* The relatively large number of *Candida parapsilosis *found in newborns could be due to the known propensity of *Candida parapsilosis* to adhere to foreign material [17, 24, 25]. It has been suggested that the high prevalence of *Candida parapsilosis* reflects the aggressive use of intravascular devices in NICUs. There is evidence that *Candida parapsilosis* is commonly carried on the hands of health care workers [25]. Transmission from infected mothers is unlikely to account for the unusual prevalence of this organism in neonates, since *Candida parapsilosis* is rarely seen on vaginal cultures, whereas *Candida glabrata,* a common vaginal isolate, is an uncommon cause of candidemia in infants and children [26, 27]. We did not identify any cases of invasive neonatal candidiasis due to *Candida krusei*. Other species, such as *Candida lusitaniae* and *Candida tropicalis,* were uncommon in our study population, as observed by others [1]. It should be noted that fluconazole prophylaxis was not used in our study population.

The previous use of a third-generation cephalosporin antibiotic was associated with mortality in our study population. In our study population, these agents (cefotaxime and ceftazidime) were used empirically in virtually all cases. Third-generation cephalosporins have been previously identified as risk factors for acquisition of an invasive fungal pathogen [6, 10, 11, 17]. Duration of prior antibacterial therapy was doubled in our nonsurvivor population. This is consistent with previous observations [17, 28]. The number of positive fungal cultures, time in the NICU, and total time of mechanical ventilation were all increased in nonsurvivors. The relationship between multiple positive cultures and outcome has been reported previously [29, 30]. Several other studies have illustrated the link between duration of intubation and length of NICU hospitalization with emergence of fungal sepsis [8, 9, 31]. 

The presence of suggestive imaging studies and multiple positive fungal cultures were predictive of mortality in infants with fungal sepsis in our population. End-organ involvement and multiple positive cultures have been shown to be markers for treatment failure and predictors of disease severity [29, 31]. Our study had the unique feature of an unusually high rate of autopsies performed, which confirmed the presence of gross pathology findings of invasive fungal disease in 5 neonates, including ischemic fungal colitis in 2 patients, peritoneal microabscesses in one patient, renal mycetomas in one neonate, and multiple organ involvement in one infant. 

These data have the limitations inherent to a single-center, retrospective study, therefore we were unable to answer questions about the reasons for delay in initiation of antifungal therapy. However, given the degree of difficulty in the diagnosis of neonatal candidiasis, its increasing incidence, and the delay of fungal cultures, we believe that a risk-factor-based approach to empirical treatment could be justified in a sick appearing newborn.

## Figures and Tables

**Table 1:** Mortality risk factors: comparison between nonsurvivor and survivor groups.

	Nonsurvivors *n* = 22	Survivors *n* = 41	*P* value
Number of positive fungal cultures, mean	5.0	2.6	0.001
Platelet count nadir during IFI: 1000 cells/mm^3^, mean	38	73	0.01
NICU stay at time of first positive fungal culture (mean, days)	69.5	32.8	0.004
Postconceptional age at time of positive culture (mean, weeks)	35.1	40.5	0.03
Gestational Age			
≤26	10	19	
27–32	1	6	
33–37	6	5	
Term	5	11	
Gender			
Male	13	17	
Female	9	24	
Congenital heart disease			
Yes	5	10	
No	17	31	
Age (days) at time of first positive fungal culture			
≤14	4	14	
15–28	4	10	
29–60	4	12	
61–120	6	4	
>120	4	1	
Time on mechanical ventilation before first positive fungal culture (mean, days)	46.0	21.6	0.002
Time with CVC before first positive fungal culture (mean, days)	60.1	31.1	0.001
Time with urinary catheter before first positive fungal culture (mean, days)	7.5	14.4	0.06
Time between first positive culture and initiation of therapy (median, days)	4	2	0.001

**Table 2:** Antibacterial use: comparison between mortality and survivor groups.

	Nonsurvivors *n* = 22	Survivors *n* = 41	*P* value
Days on antibacterial drugs before first positive fungal culture: mean	36.3	17.3	0.002
Total days on antibacterial drugs: mean	63.9	40	0.006
Antibacterial agents used before invasive fungal infection			
Glycopeptides (vancomycin) (%)	20 (91)	28 (68)	0.06
Aminoglycosides (amikacin, gentamicin, tobramycin) (%)	18 (82)	29 (71)	0.34
3rd generation cephalosporins (cefotaxime, ceftazidime) (%)	21 (95)	26 (63)	0.003
Carbapenem (imipenem/cilastatin, meropenem) (%)	8 (36)	8 (19.5)	0.16
Anaerobic (clindamycin, metronidazole) (%)	6 (27)	6 (15)	0.32

**Table 3:** Origin of positive culture specimen: comparison between mortality and survivor groups.

Culture site	Nonsurvivors *n* = 18 (%)	Survivors *n* = 33 (%)	*P* value
Blood (only isolate)	3 (17)	9 (50)	0.52
Blood (single or multiple isolates)	9 (50)	15 (45)	0.79
Tracheal aspiration (only)	4 (18)	8 (20)	1.0
Urine catheterization (only)	2 (11)	9 (27)	0.30
Peritoneal or pleural fluid (only)	2 (11)	3 (17)	1.0
2 or more positive sites	11 (61)	12 (36)	0.17
